# Supplementary material for: Mechanistic insights into C-C coupling in electrochemical CO reduction using gold superlattices
Source: Nat Commun. 2024 Jan 24;15:720. doi: 10.1038/s41467-024-44923-x (PMC10808111; doi:10.1038/s41467-024-44923-x)
Supplement: Supplementary file 1 — Supplementary Information [file 41467_2024_44923_MOESM1_ESM.pdf]

Supplementary Information

Mechanistic Insights into C-C Coupling in Electrochemical CO Reduction using Gold Superlattices

Xiaoju Yang,[1,2,‡] Chao Rong,[3,‡] Li Zhang,[1,2] Zhenkun Ye,[1,2] Zhiming Wei,[4] Chengdi Huang,[1,2] Qiao Zhang,[4] Qing Yuan,[1,2] Yueming Zhai,[4] Fu-Zhen Xuan,[3] Bingjun Xu,[5,*] Bowei Zhang,[3,*] and Xuan Yang[1,2,*]

[1]Key Laboratory of Material Chemistry for Energy Conversion and Storage, Huazhong University of Science and Technology, Wuhan 430074, China

[2]Hubei Key Laboratory of Bioinorganic Chemistry and Materia Medica, School of Chemistry and Chemical Engineering, Huazhong University of Science and Technology, Wuhan 430074, China

[3]Shanghai Key Laboratory of Intelligent Sensing and Detection Technology, School of Mechanical and Power Engineering, East China University of Science and Technology, Shanghai 200237, China

[4]The Institute for Advanced Studies, Wuhan University, Wuhan 430072, China

[5]College of Chemistry and Molecular Engineering, Peking University, Beijing 100871, China

[‡]These authors contributed equally to this work

*Corresponding authors: b_xu@pku.edu.cn, boweiz@ecust.edu.cn, and xuanyang@hust.edu.cn

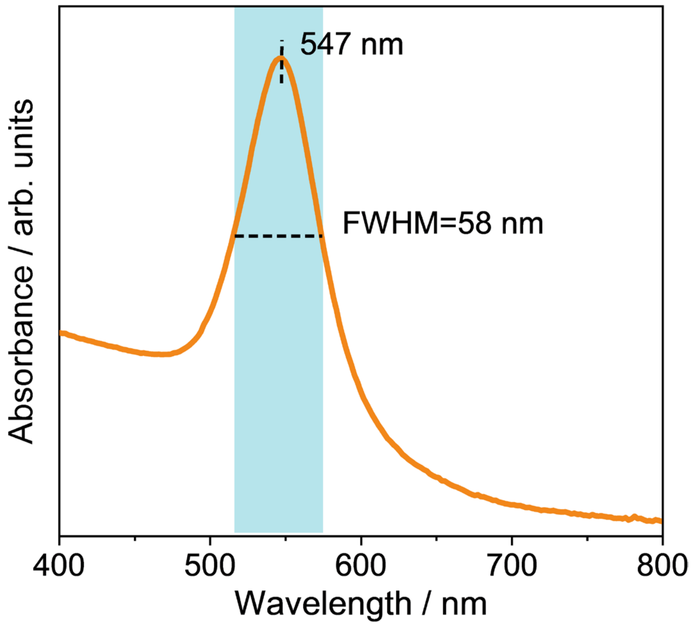

Supplementary Fig. 1 | UV−vis spectrum of Au nanocubes with an average edge length of 40 nm. The LSPR peak of Au nanocubes is located at around 547 nm and the full width at half maximum was around 58 nm, suggesting a homogeneous distribution for the gold nanocubes.

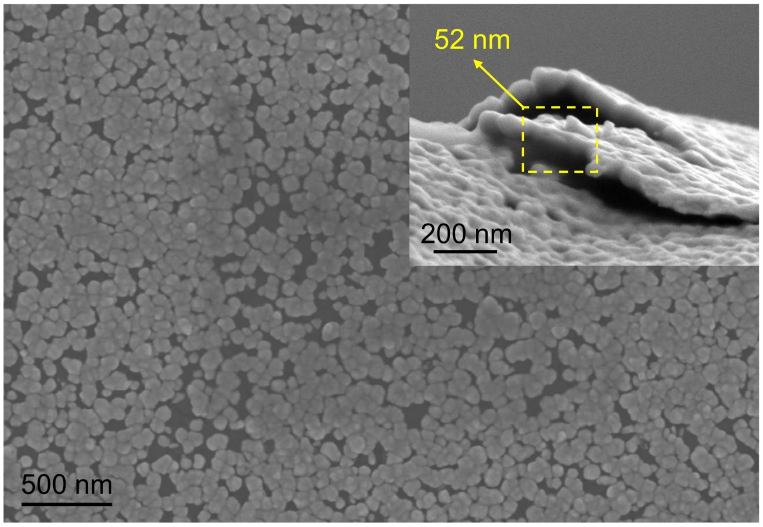

Supplementary Fig. 2 | A typical SEM image of CDFs with an average thickness of 52 nm before reaction. Traditional CDFs shows an irregularly ordered structure.

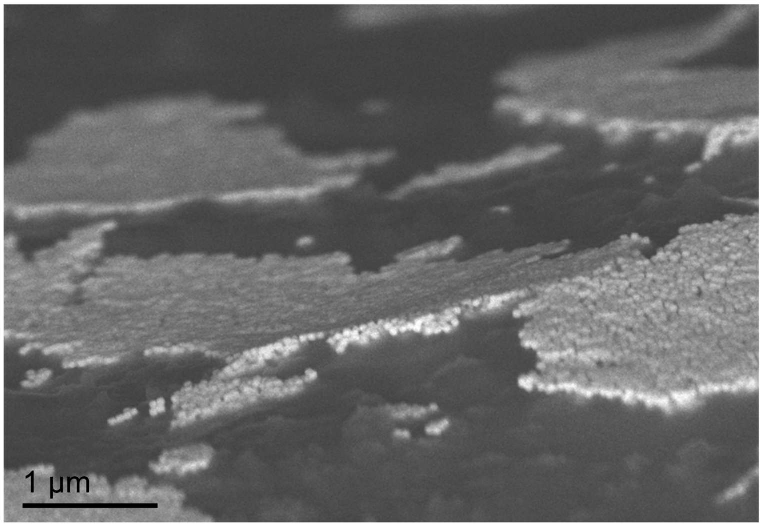

Supplementary Fig. 3 | A typical SEM image of GNSs with a monolayer of gold nanocubes before reaction. The thickness of GNSs is around 40 nm.

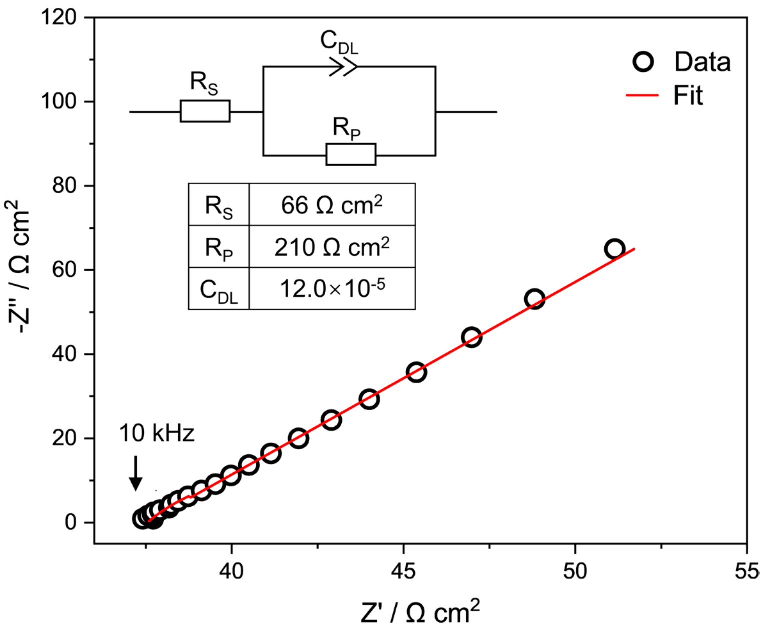

Supplementary Fig. 4 | Electrochemical impedance spectroscopy of CDFs. The R_S of CDFs is determined to be 66 Ω cm^2.

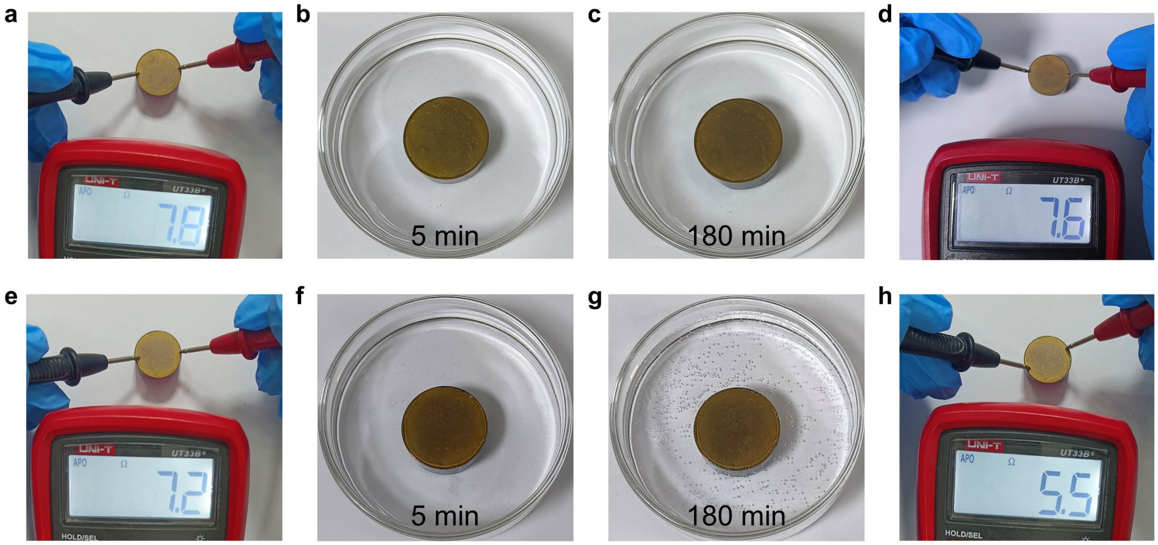

Supplementary Fig. 5 | The stability testing of GNSs immersed in 0.1 M $HClO_4$ (a−d) and 0.5 M $KHCO_3$ (e−h), respectively. Corresponding resistances recorded before (**a**, **e**) and after (**d**, **h**) immersed in the electrolytes for 180 min.

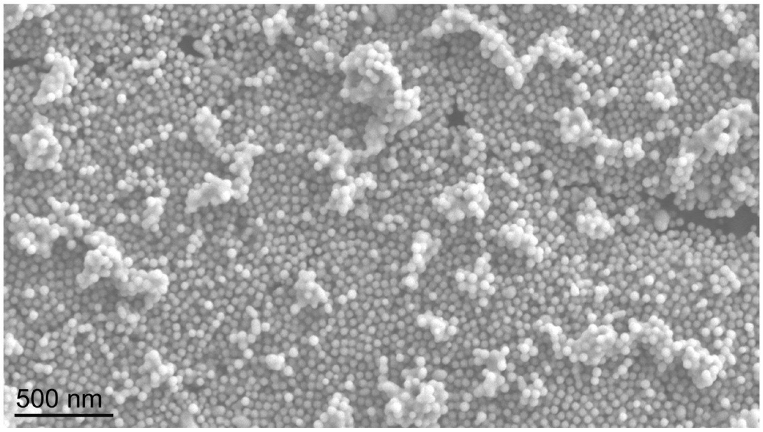

Supplementary Fig. 6 | A typical SEM image of GNSs after CV measurements. The rhombic structure of the superlattices and morphology of gold nanocubes are mostly well preserved.

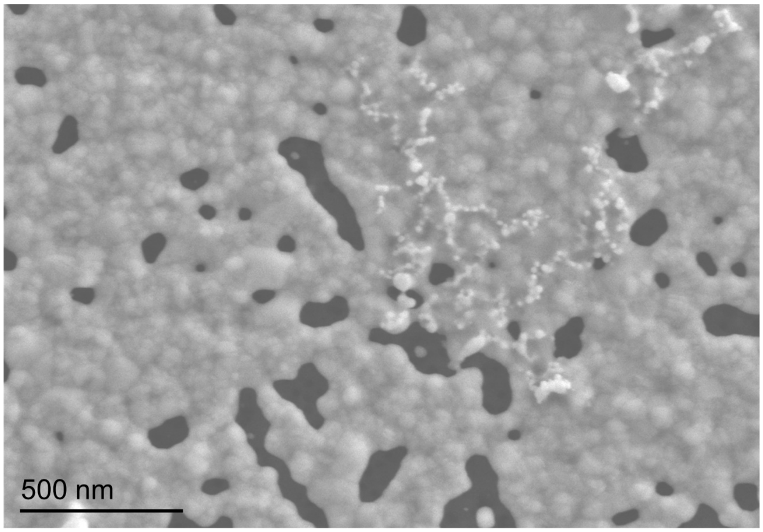

Supplementary Fig. 7 | A typical SEM image of CDFs after CV measurements. The morphology of CDFs changes significantly.

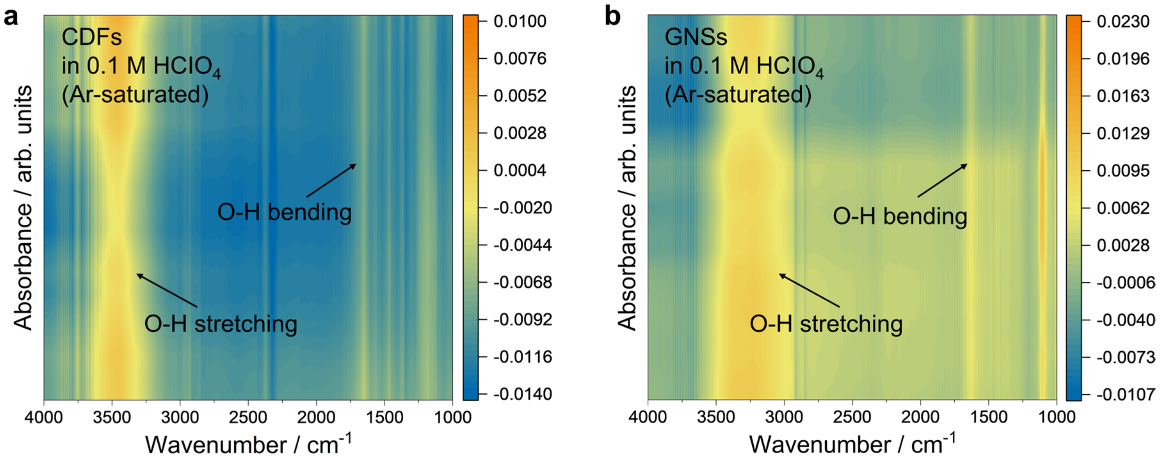

Supplementary Fig. 8 | *In situ* SEIRA spectra collected on different films. The SEIRA spectra collected on the surfaces of **a** CDFs and **b** GNSs in the potential range from 1.0 to −0.8 V (from bottom to top) in Ar-saturated 0.1 M $HClO_4$.

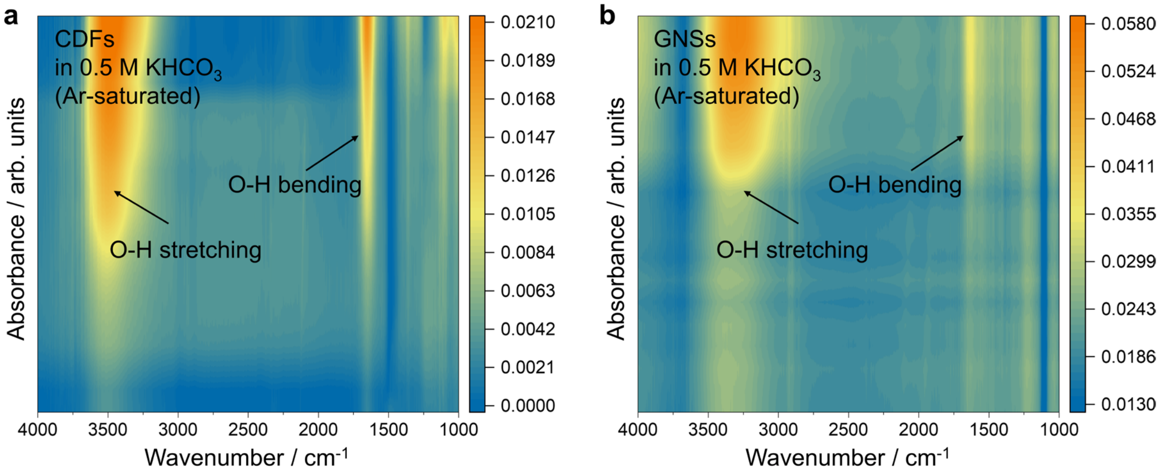

Supplementary Fig. 9 | *In situ* SEIRA spectra collected on different films. The SEIRA spectra on the surfaces of **a** CDFs and **b** GNSs in the potential range from 1.0 to −0.8 V (from bottom to top) in Ar-saturated 0.5 M $KHCO_3$.

The behaviors of H_2O stretching (3600−3000 cm^{-1}) and bending modes (~1640 cm^{-1}) on the surfaces of GNSs are consistent to those on CDFs in acidic and neutral electrolytes,[1,2] indicating that GNSs are potential alternative substrates for *in situ* ATR-SEIRAS.

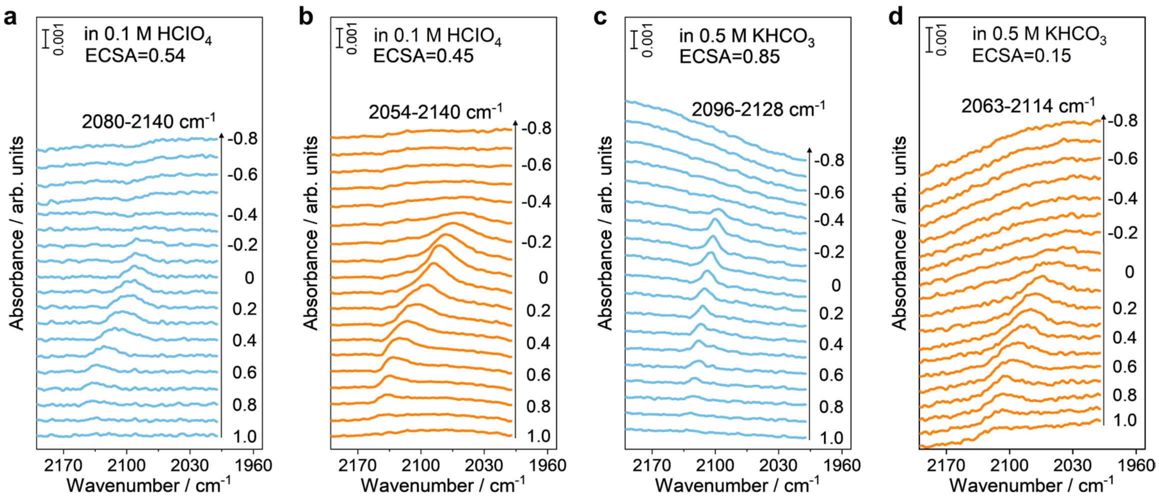

Supplementary Fig. 10 | Potential-dependent SEIRA spectra of adsorbed CO bands in 0.1 M $HClO_4$ (a, b) and 0.5 M $KHCO_3$ (c, d). Blue and orange lines represent CDFs and GNSs, respectively.

The behaviors of adsorbed CO bands (~2080 cm^{-1}) on the surfaces of GNSs are consistent to those on CDFs.[3,4]

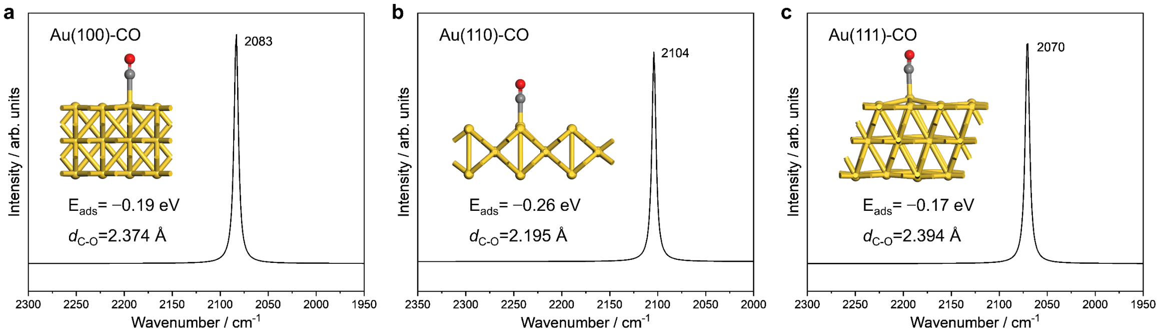

Supplementary Fig. 11 | Simulated IR spectra of CO adsorptions on the surfaces of Au with different facets: a Au(100), b Au(110), and c Au(111). The insets in **a**, **b**, and **c** show the adsorption configurations, adsorption energy, and the bond length of CO.

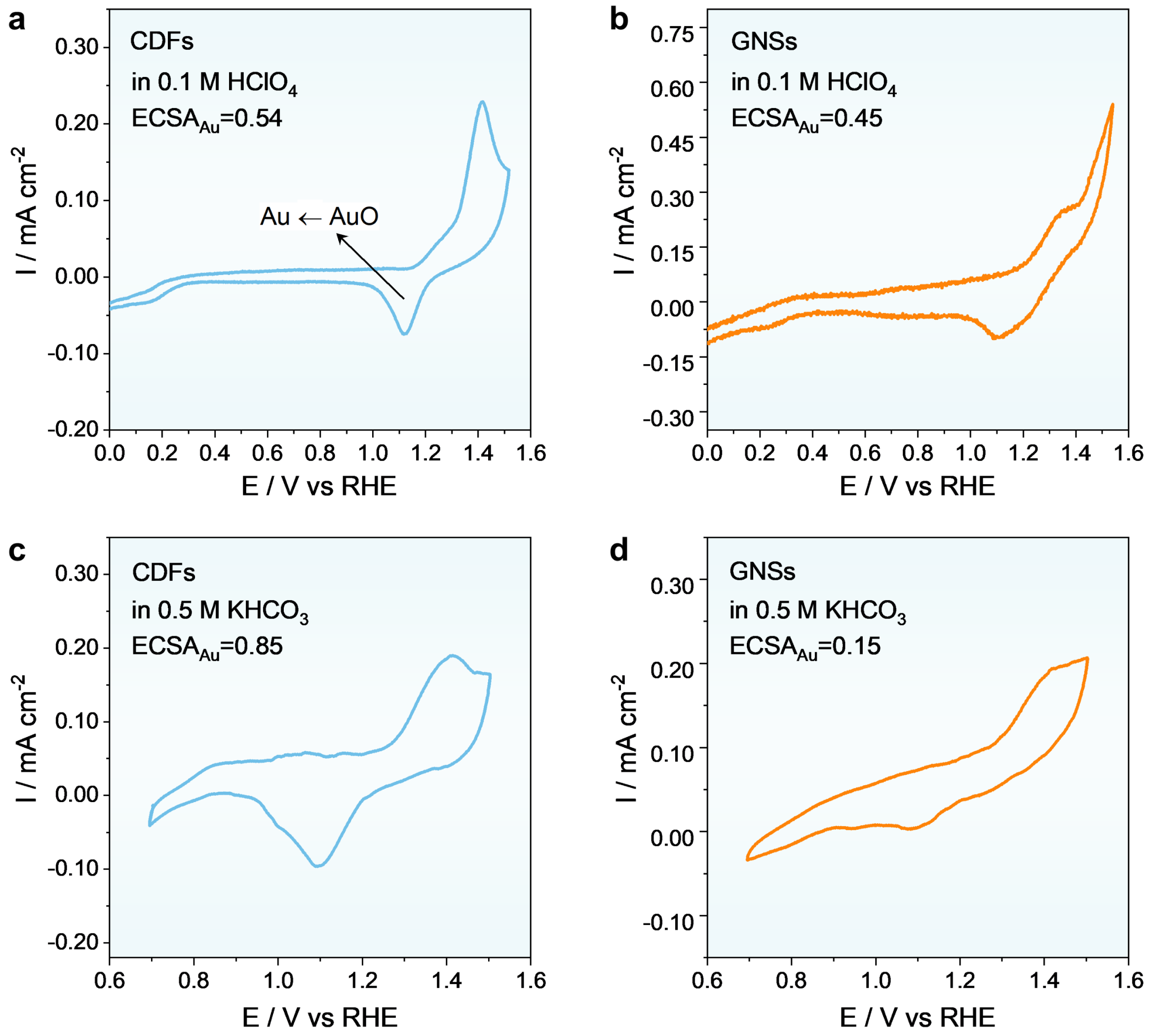

Supplementary Fig. 12 | Cyclic voltammograms recorded on the surfaces of CDFs and GNSs in different electrolytes: a, **b** Ar-saturated 0.1 M $HClO_4$, **c**, **d** Ar-saturated 0.5 M $KHCO_3$.

Supplementary Table 1. The peak areas of CO adsorptions on CDFs and GNSs in the potential range from 0.9 to −0.3 V in 0.1 M $HClO_4$.

Substrate / E / V vs RHE	CDFs	GNSs
0.9	0.00145	0.00280
0.8	0.00264	0.00540
0.7	0.00489	0.01170
0.6	0.01140	0.01454
0.5	0.01409	0.02570
0.4	0.02474	0.03350
0.3	0.02552	0.05440
0.2	0.03196	0.07790
0.1	0.02449	0.09990
0	0.01418	0.12470
−0.1	0.00877	0.09570
−0.2	0.00498	0.09080
−0.3	0.00496	0.07270

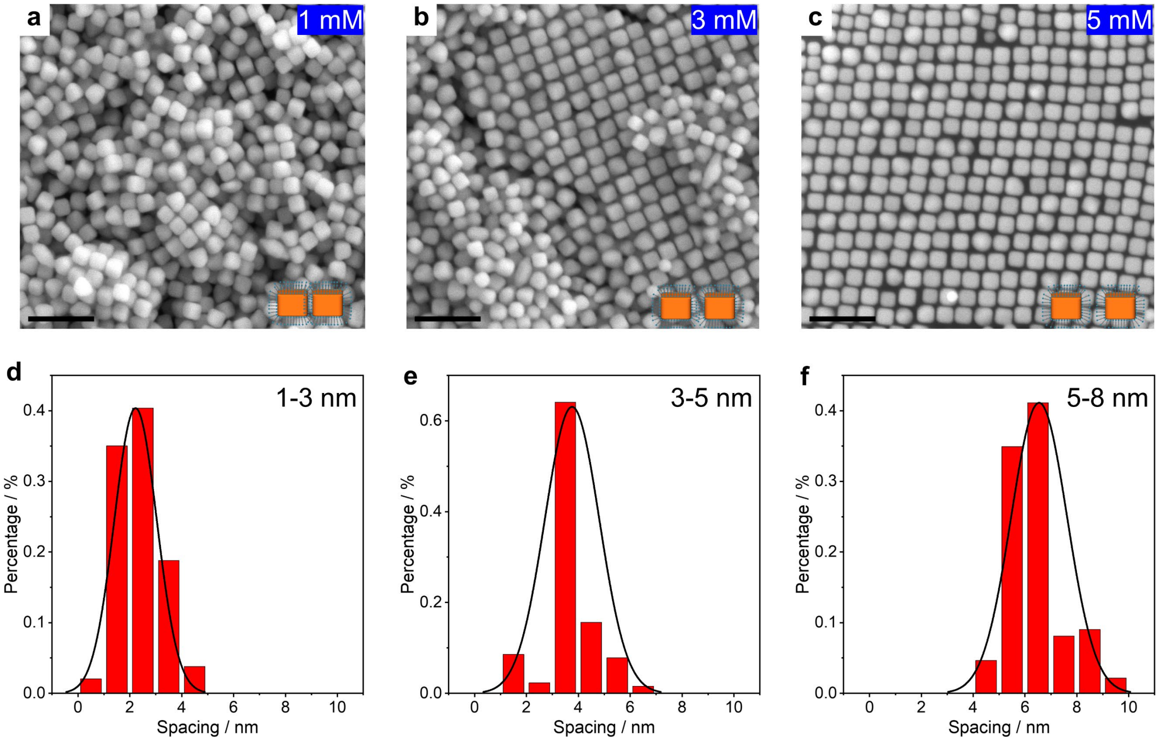

Supplementary Fig. 13 | The SEM images of SAFs (a−c) fabricated in the presence of different concentrations of CPC and the corresponding gap distributions of Au nanocubes (d−f). The scale bar is 200 nm.

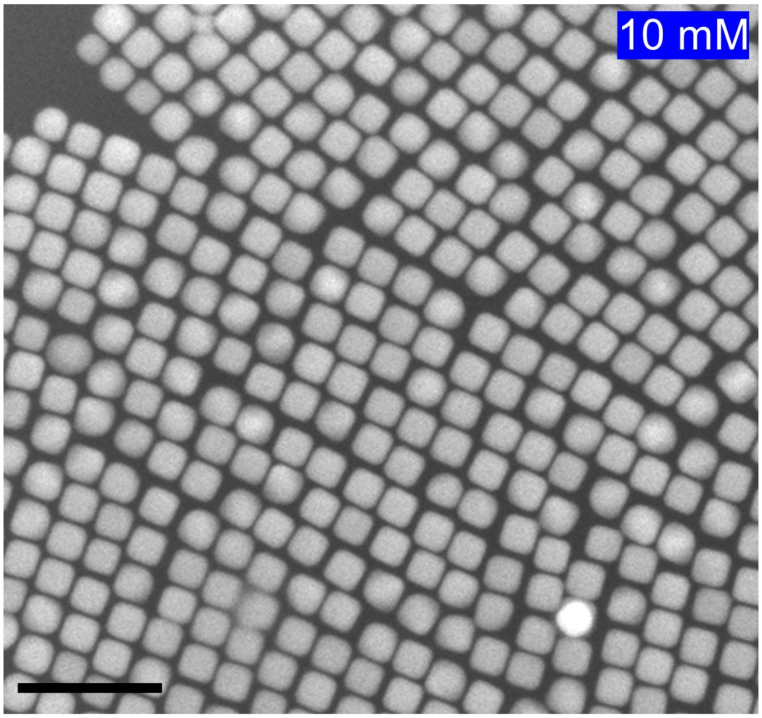

Supplementary Fig. 14 | A typical SEM image of SAFs fabricated in the presence of 10 mM CPC. The scale bar is 200 nm.

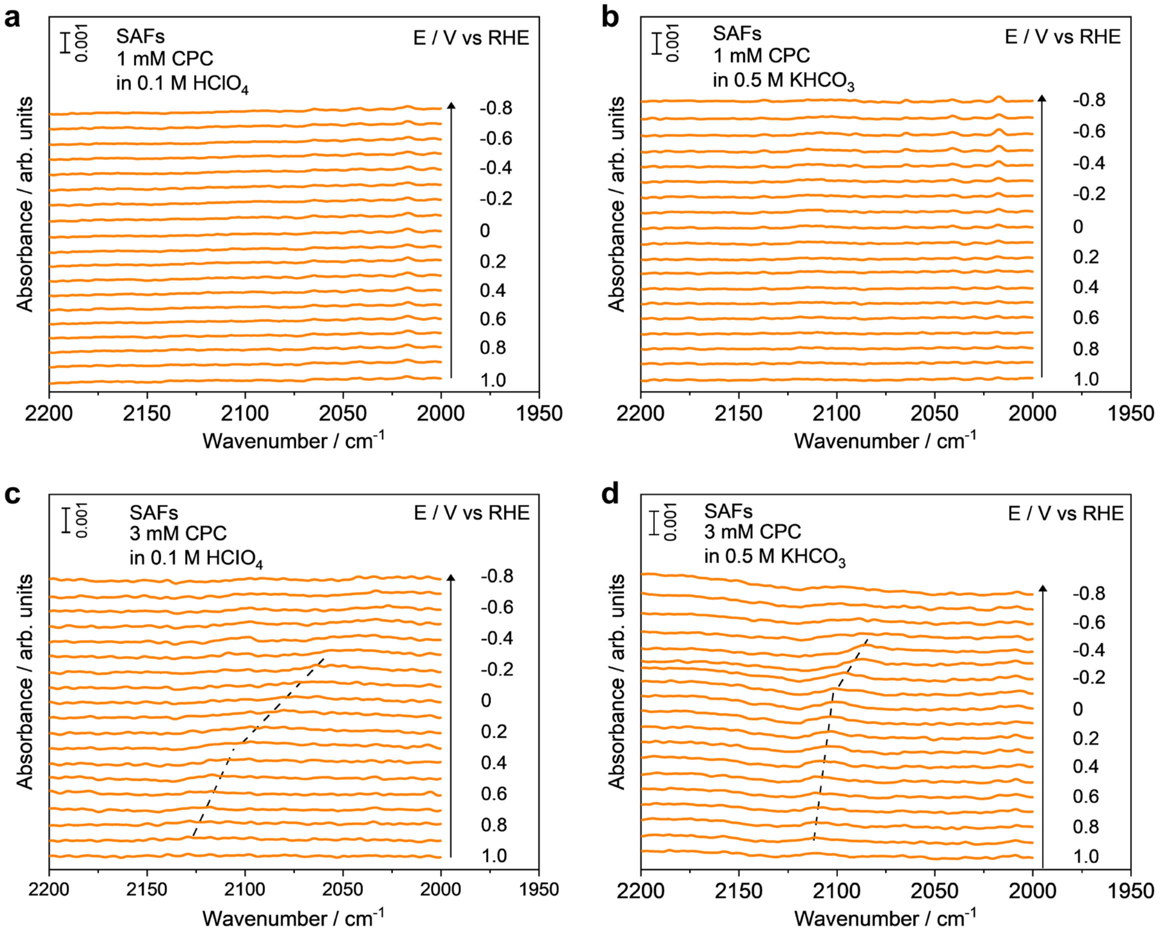

Supplementary Fig. 15 | Potential-dependent SEIRA spectra of adsorbed CO bands on the surfaces of SAFs fabricated in the presence of 1 mM (a, b) and 3 mM (c, d) CPC. a, c In CO-saturated 0.1 M $HClO_4$. **c, d** In CO-saturated 0.5 M $KHCO_3$.

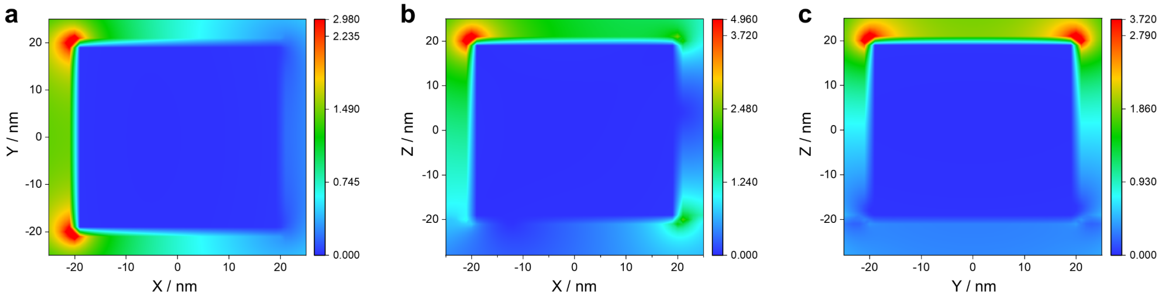

Supplementary Fig. 16 | FDTD simulation showing the intensity of the C≡O vibration (~2100 cm^{-1}) on a single Au nanocube (40×40×40 nm). a XY plane, **b** XZ plane, and **c** YZ plane.

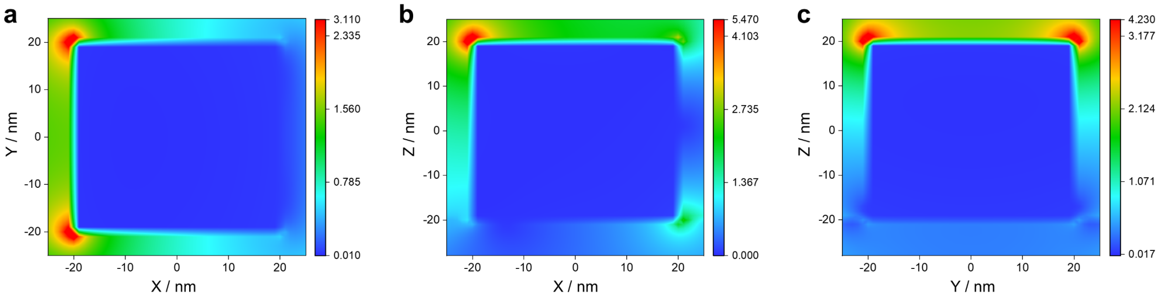

Supplementary Fig. 17 | FDTD simulation showing the intensity of the O-H stretching vibration (3200−3400 cm^{-1}) on a single Au nanocube (40×40×40 nm). a XY plane, **b** XZ plane, and **c** YZ plane.

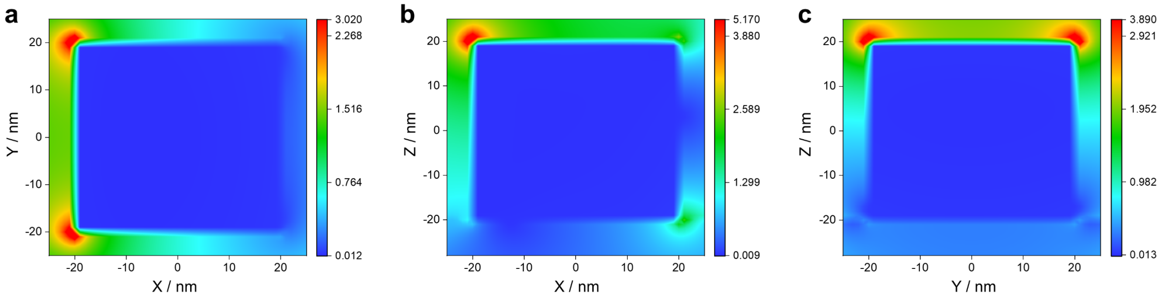

Supplementary Fig. 18 | FDTD simulation showing the intensity of the C-H vibration (~2800 cm^{-1}) on a single Au nanocube (40×40×40 nm). a XY plane, **b** XZ plane, and **c** YZ plane.

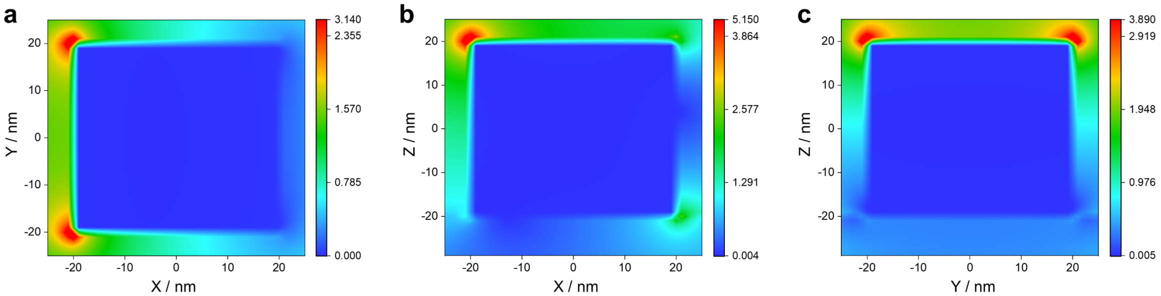

Supplementary Fig. 19 | FDTD simulation showing the intensity of the C=O vibration (~1750 cm^{-1}) on a single Au nanocube (40×40×40 nm). a XY plane, **b** XZ plane, and **c** YZ plane.

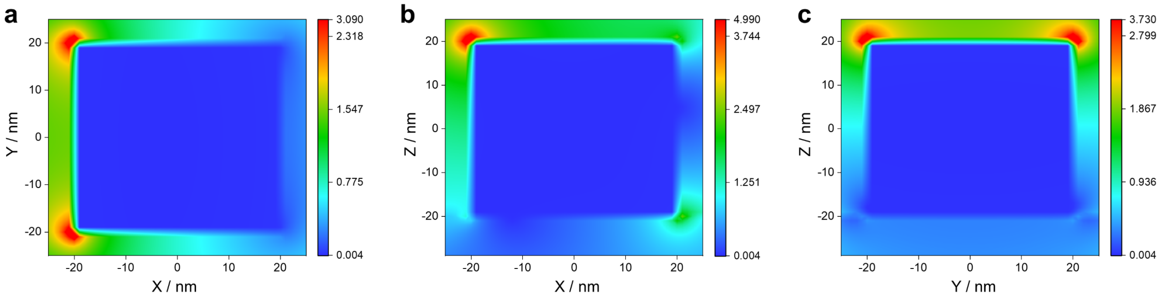

Supplementary Fig. 20 | FDTD simulation showing the intensity of the O-H bending vibration (~1650 cm^{-1}) on a single Au nanocube (40×40×40 nm). a XY plane, **b** XZ plane, and **c** YZ plane.

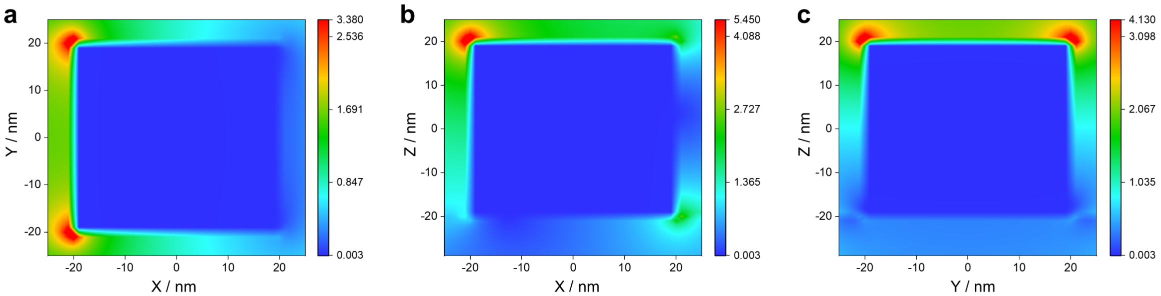

Supplementary Fig. 21 | FDTD simulation showing the intensity of the C-O bending vibration (~1300 cm^{-1}) on a single Au nanocube (40×40×40 nm). a XY plane, **b** XZ plane, and **c** YZ plane.

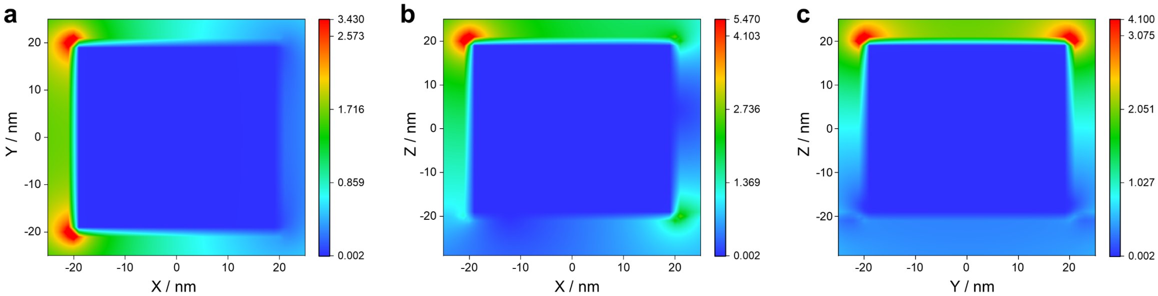

Supplementary Fig. 22 | FDTD simulation showing the intensity of the O_2^- vibration (~1100 cm^{-1}) on a single Au nanocube (40×40×40 nm). a XY plane, **b** XZ plane, and **c** YZ plane.

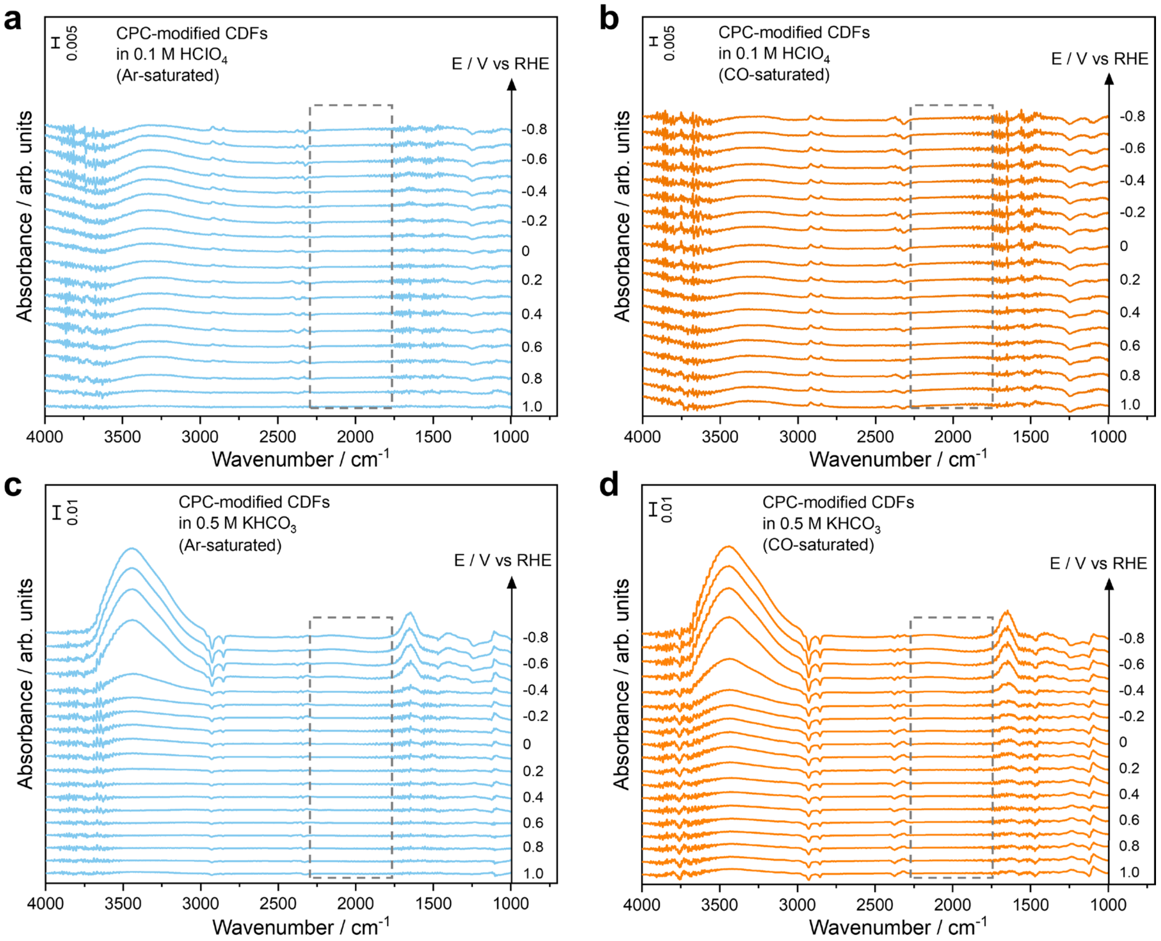

Supplementary Fig. 23 | Potential-dependent SEIRA spectra on the surfaces of CPC-modified CDFs (the concentration of CPC is 5 mM) in different electrolytes: a Ar-saturated 0.1 M $HClO_4$, **b** CO-saturated 0.1 M $HClO_4$, **c** Ar-saturated 0.5 M $KHCO_3$, and **d** CO-saturated 0.5 M $KHCO_3$.

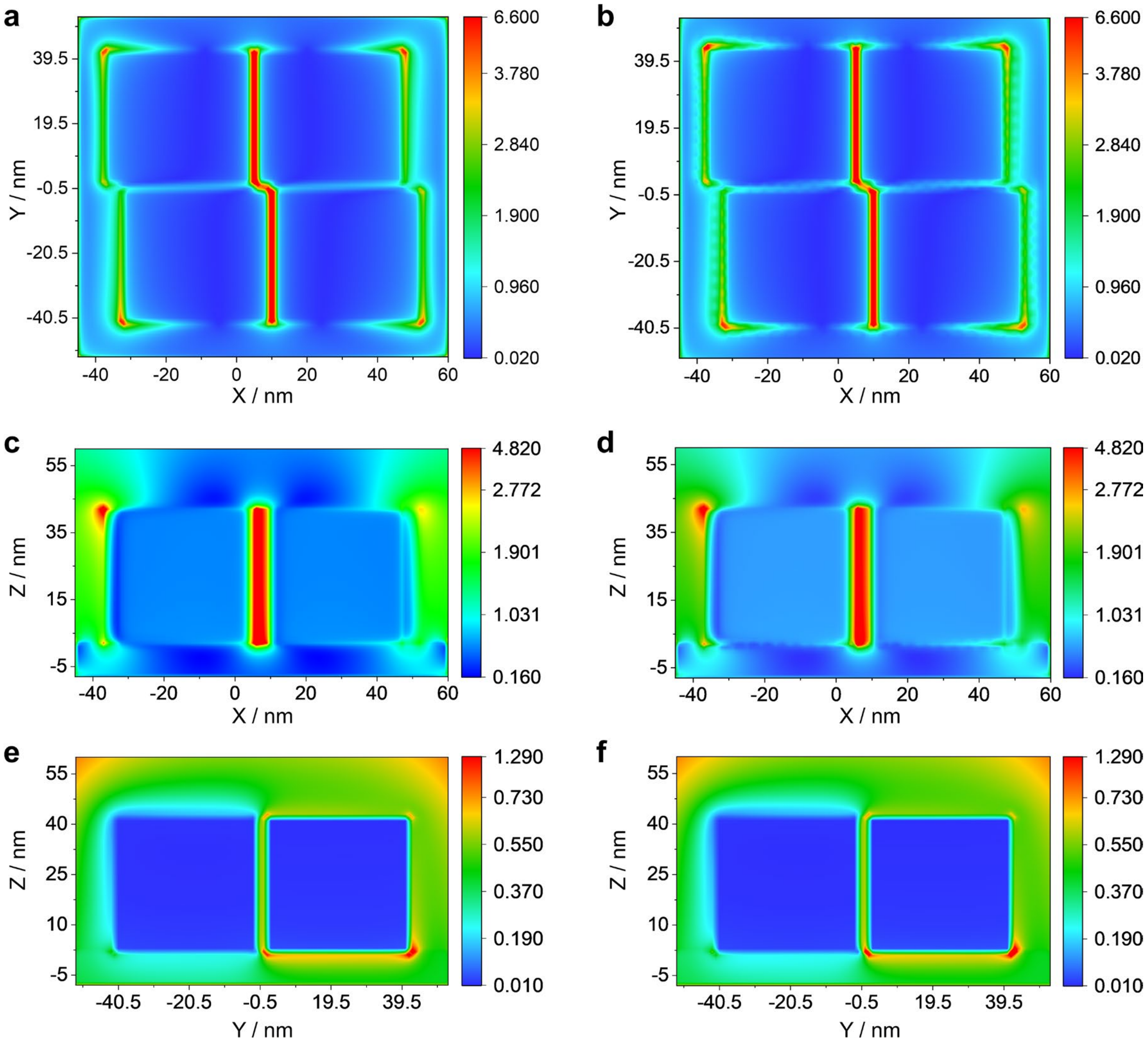

Supplementary Fig. 24 | FDTD simulations of the local electromagnetic field for GNSs before (a, c, and e) and after (b, d, and f) the modification of CPC. Simulated near-field enhancement $|E|^2$ on XY planes (**a** and **b**), XZ planes (**c** and **d**), and YZ planes (**e** and **f**).

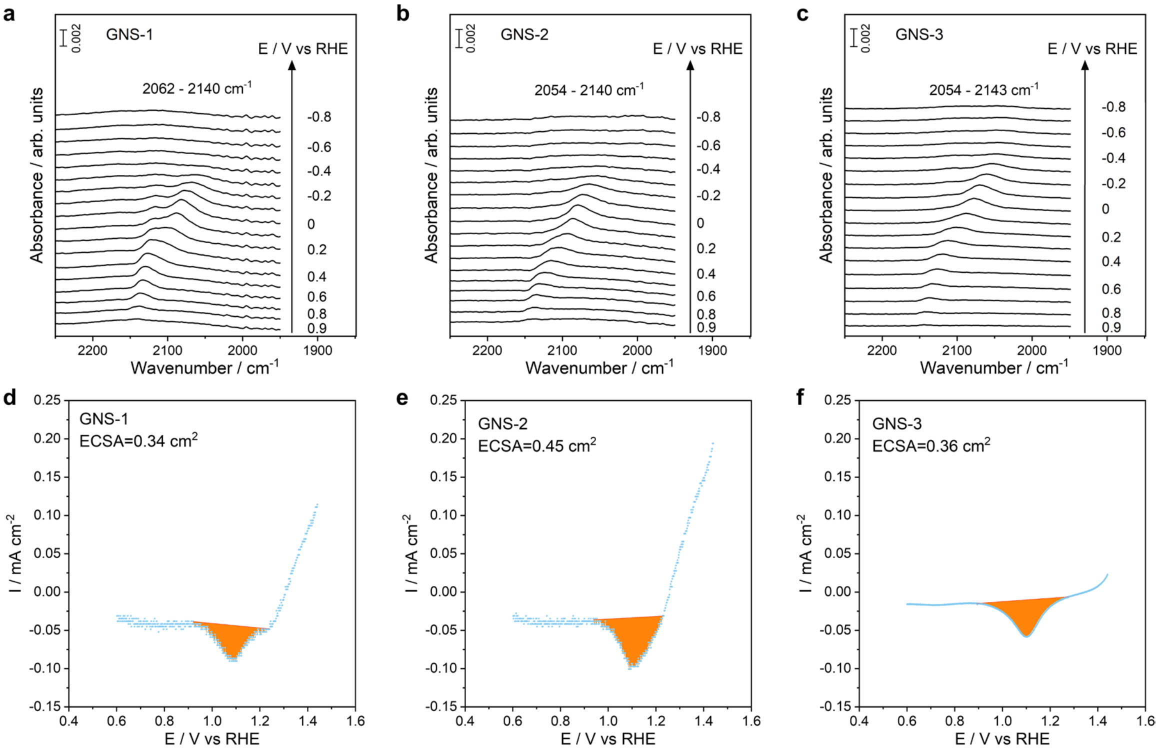

Supplementary Fig. 25 | Potential-dependent SEIRA spectra of adsorbed CO bands on three different GNSs in CO-saturated 0.1 M $HClO_4$: a GNS-1, **b** GNS-2, and **c** GNS-3. Cyclic voltammograms showing the ECSAs of GNSs: **d** GNS-1, **e** GNS-2, and **f** GNS-3.

Supplementary Table 2. The maximum peak areas of CO adsorptions, ECSAs, and REFs of three different GNSs: GNS-1, GNS-2, and GNS-3.

	Maximum peak area of CO adsorptions	ECSAs / cm^2	REFs
GNS-1	0.1003	0.34	0.296
GNS-2	0.1247	0.45	0.274
GNS-3	0.1060	0.36	0.291
Relative standard deviation (RSD)	11.57%	15.29%	4.02%

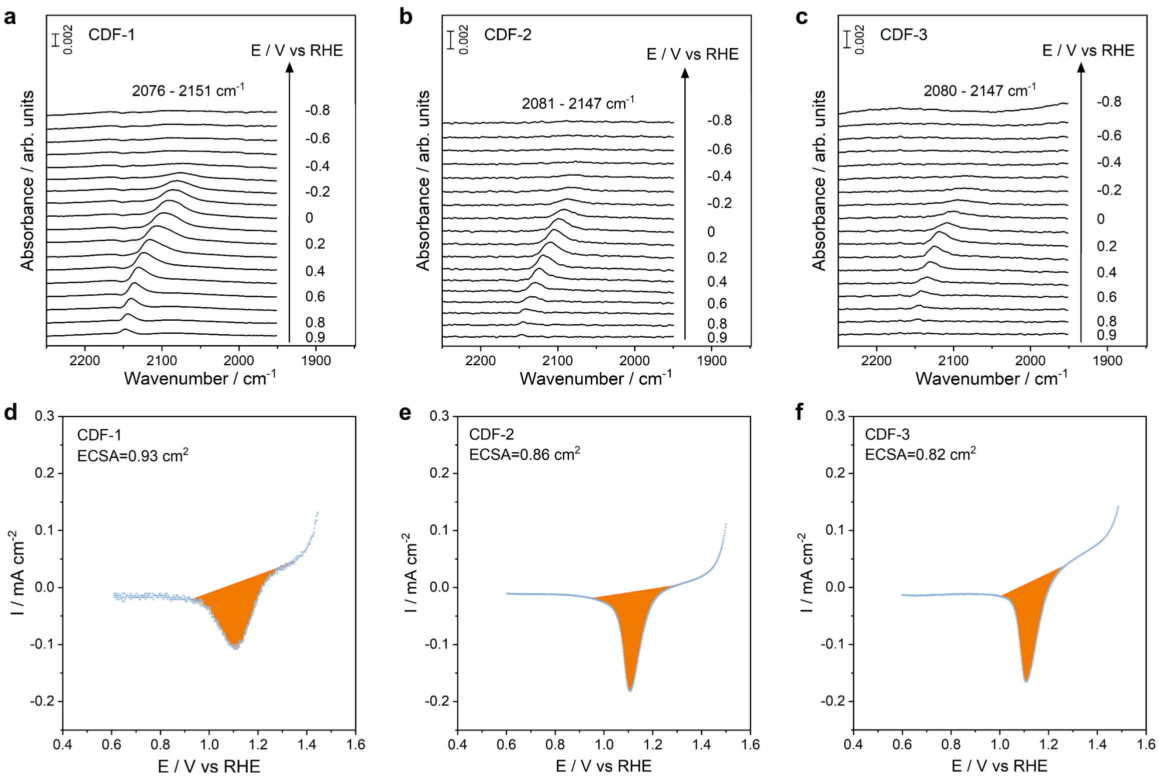

Supplementary Fig. 26 | Potential-dependent SEIRA spectra of adsorbed CO bands on three different CDFs in CO-saturated 0.1 M $HClO_4$: a CDF-1, **b** CDF-2, and **c** CDF-3. Cyclic voltammograms showing the ECSAs of CDFs: **d** CDF-1, **e** CDF-2, and **f** CDF-3.

Supplementary Table 3. The maximum peak areas of CO adsorptions, ECSAs, and REFs of three different CDFs: CDF-1, CDF-2, and CDF-3.

	Maximum peak area of CO adsorptions	ECSAs / cm^2	REFs
CDF-1	0.1064	0.93	0.144
CDF-2	0.0500	0.86	0.058
CDF-3	0.0348	0.82	0.042
Relative standard deviation (RSD)	59.19%	6.40%	67.45%

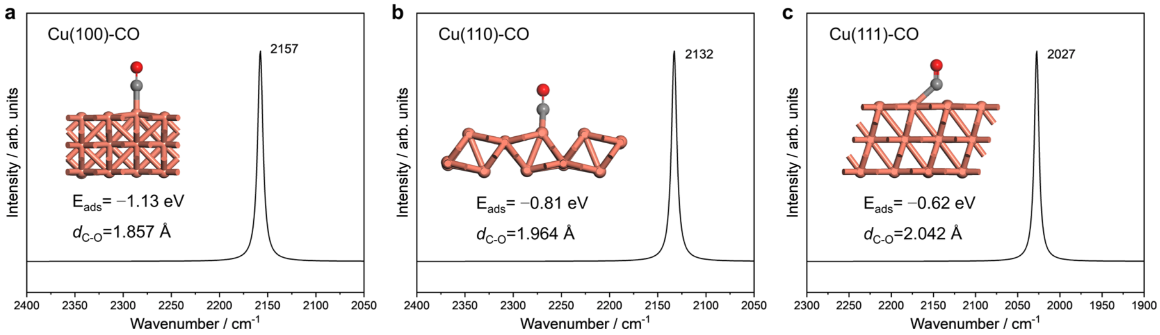

Supplementary Fig. 27 | Simulated IR spectra of CO adsorptions on the surfaces of Cu with different facets: a Cu(100), b Cu(110), and c Cu(111). The insets in **a**, **b**, and **c** show the adsorption configurations, adsorption energy, and the bond length of CO.

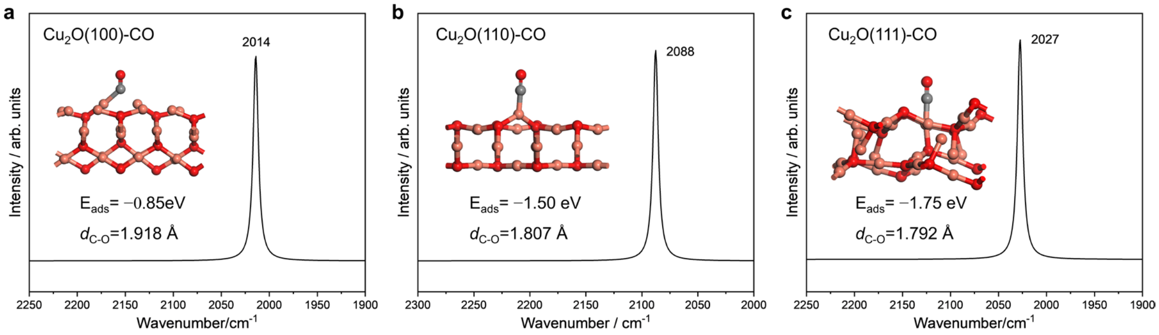

Supplementary Fig. 28 | Simulated IR spectra of CO adsorptions on the surfaces of Cu_2O with different facets: a Cu_2O(100), b Cu_2O(110), and c Cu_2O(111). The insets in **a**, **b**, and **c** show the adsorption configurations, adsorption energy, and the bond length of CO.

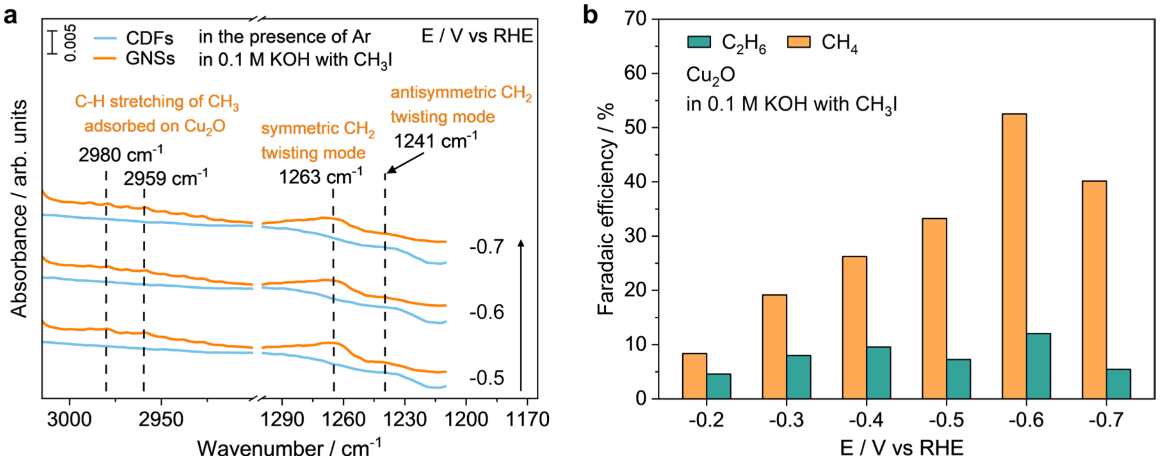

Supplementary Fig. 29 | SEIRA spectra of the intermediates and Faradaic efficiencies for methane and ethane. a Potential-dependent SEIRA spectra on different substrates in Ar-saturated 0.1 M KOH with CH_3I. **b** Faradaic efficiencies of methane and ethane in Ar-saturated 0.1 M KOH with CH_3I on the surfaces of Cu_2O catalysts.

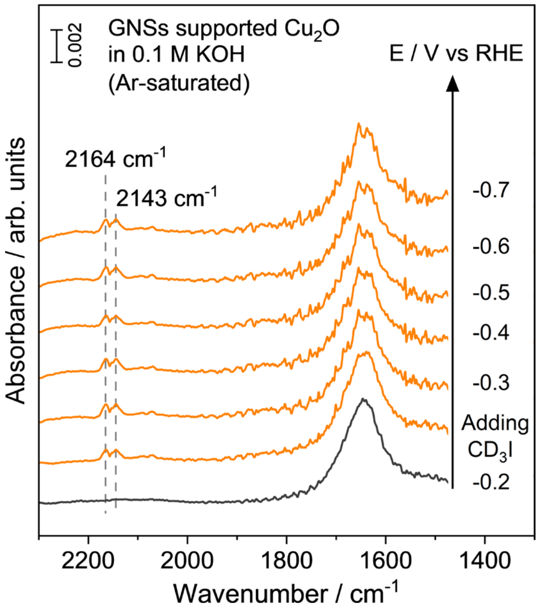

Supplementary Fig. 30 | Potential-dependent SEIRA spectra on the surfaces of GNSs supported Cu_2O in Ar-saturated 0.1 M KOH with CD_3I. Grey and orange lines represent the SEIRA spectra collected before and after the addition of CD_3I, respectively.

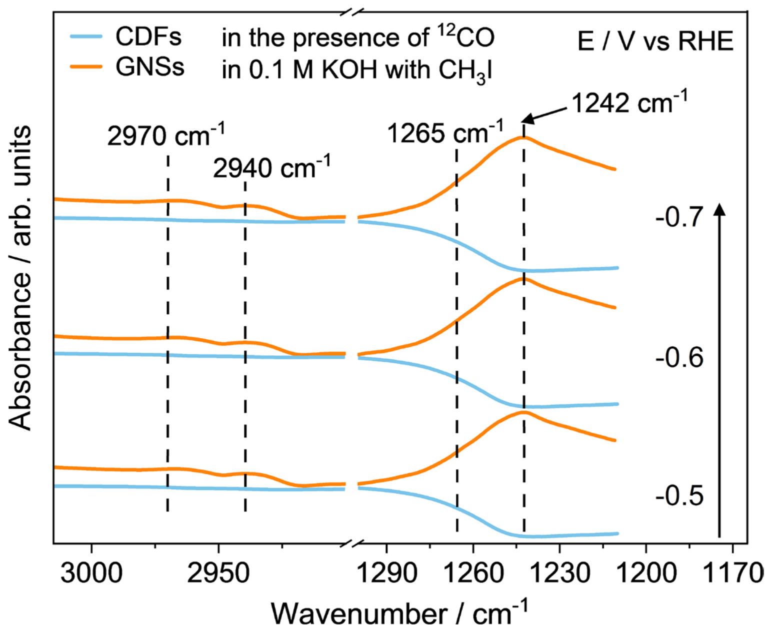

Supplementary Fig. 31 | Potential-dependent SEIRA spectra on different substrates in ^{12}CO-saturated 0.1 M KOH with CH_3I. Blue and orange lines represent the SEIRA spectra collected on CDFs and GNSs, respectively.

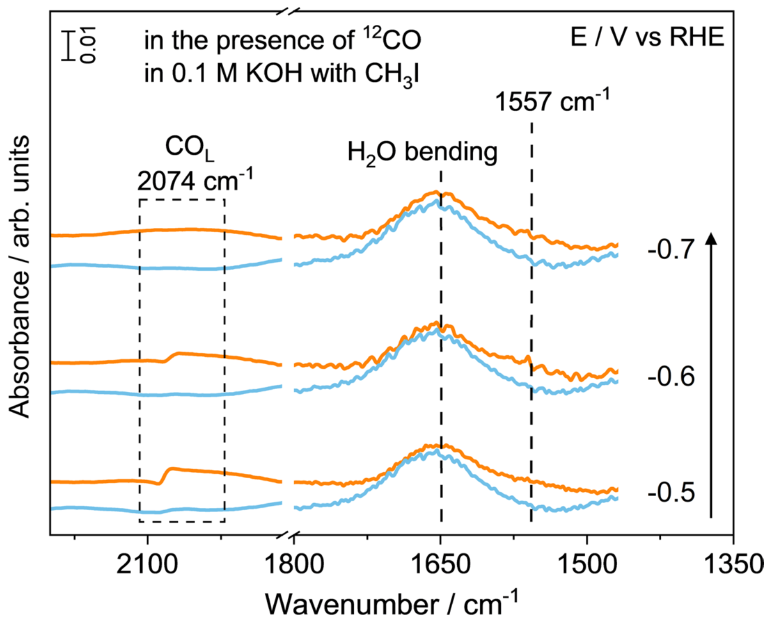

Supplementary Fig. 32 | SEIRA spectra of CO_L and *OCCOH vibrations in ^{12}CO-saturated 0.1 M KOH with CH_3I. Blue and orange lines represent the SEIRA spectra collected on CDFs and GNSs, respectively.

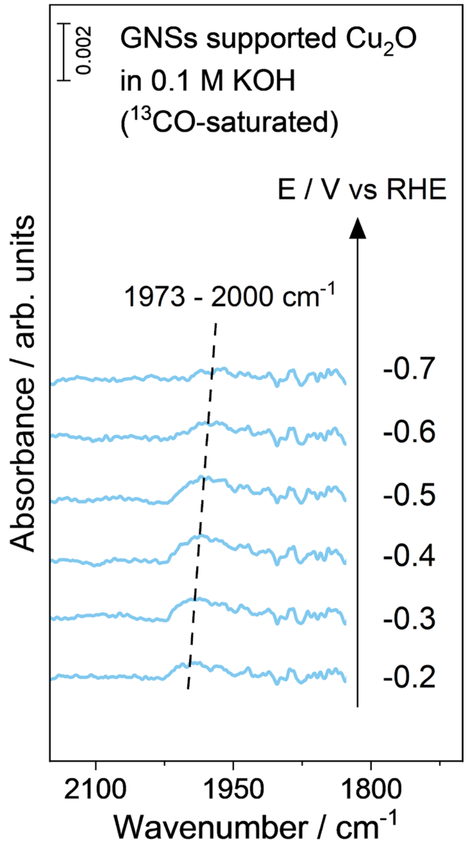

Supplementary Fig. 33 | Potential-dependent SEIRA spectra on the surfaces of GNSs supported Cu_2O in ^{13}CO-saturated 0.1 M KOH solution. The ^{13}CO adsorptions on the surfaces of GNSs supported Cu_2O locate at around 2000 cm^{-1}.

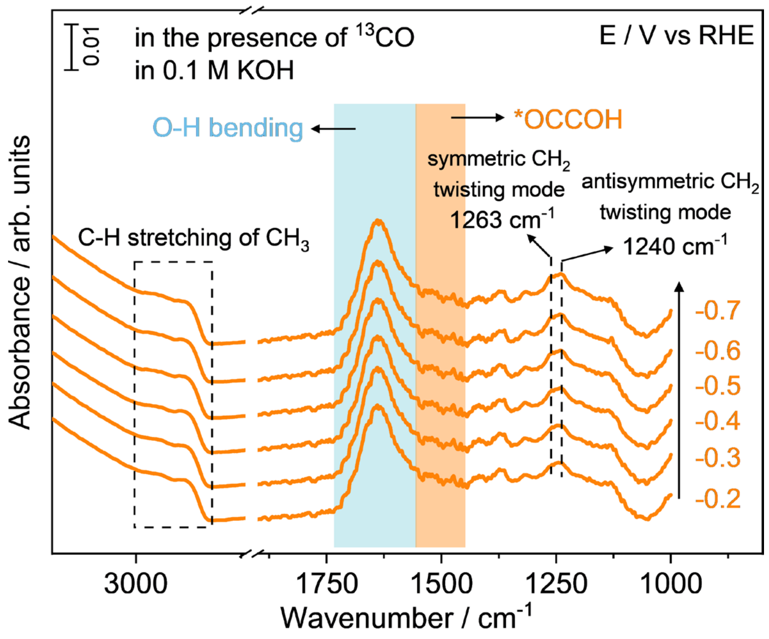

Supplementary Fig. 34 | Potential-dependent SEIRA spectra on GNSs supported Cu_2O catalysts in ^{13}CO-saturated 0.1 M KOH with CH_3I. The peak positions of C-H stretching and CH_2 twisting modes are similar to those in ^{12}CO-saturated 0.1 M KOH with CH_3I

Supplementary References

1. Miyake, H., Ye, S. & Osawa, M. Electroless deposition of gold thin films on silicon for surface-enhanced infrared spectroelectrochemistry. *Electrochem. Commun.* **4**, 973−977 (2002).

2. Dunwell, M., Yan, Y. & Xu, B. A surface-enhanced infrared absorption spectroscopic study of pH dependent water adsorption on Au. *Surf. Sci.* **650**, 51−56 (2016).

3. Chang, X. et al. Understanding the complementarities of surface-enhanced infrared and Raman spectroscopies in CO adsorption and electrochemical reduction. *Nat. Commun.* **13**, 2656−2667 (2022).

4. Chang, X. et al. Determining intrinsic stark tuning rates of adsorbed CO on copper surfaces. *Catal. Sci. Technol.* **11**, 6825−6831 (2021).
